# Moderate-to-Vigorous Physical Activity and Suicidal Ideation in Adolescents with Attention Deficit/Hyperactivity Disorder: the Mediating Effects of Mental Health

**DOI:** 10.1007/s10803-025-06809-9

**Published:** 2025-04-20

**Authors:** Chang Liu, Yijian Yang, Kelly Arbour-Nicitopoulos, Rainbow Tin-hung Ho, Juanita Sin-ting Cheung, Andes Leung, Cindy Hui-ping Sit

**Affiliations:** 1 Department of Sports Science and Physical Education, The Chinese University of Hong Kong, Shatin, New Territories, Hong Kong China; 2 Faculty of Kinesiology and Physical Education, University of Toronto, 55 Harbord Street, Toronto, ON M5S 2W6 Canada; 3 Department of Social Work & Social Administration, The University of Hong Kong, Hong Kong, China; 4 RunOurCity Foundation Limited, Hong Kong, China

**Keywords:** Anxiety, Depression, Stress, Resilience, Youth

## Abstract

This study aimed to (a) examine whether device-assessed moderate-to-vigorous physical activity (MVPA) was associated with suicidal ideation (SI) in adolescents with attention-deficit/hyperactivity disorders (ADHD), (b) investigate whether the association was mediated by depression, and (c) assess whether anxiety, stress, and resilience would work with depression to constitute chain mediation paths between MVPA and SI. Sixty adolescents with ADHD aged 12–17 (Mage = 14.33 ± 1.43 years) met the inclusion criteria. MVPA data were assessed using accelerometers worn around the waist for seven consecutive days. SI, depression, anxiety, stress, and resilience were examined using self-report questionnaires. Bivariate correlations were estimated for all variables. Lavaan package was used to examine the association between MVPA and SI, and the mediating effects of depression, anxiety, stress, and resilience by adjusting for a minimally sufficient confounder set and applying the bootstrap method. MVPA was negatively associated with SI. Depression fully mediated this association. Anxiety, stress, and resilience combining with depression formed three chain mediation paths between MVPA and SI, respectively. The integrative mediation model, including anxiety, stress, resilience, and depression, revealed that anxiety and depression explained 38.2% of the variance in the association between MVPA and SI, and resilience and depression explained 22.1%, while stress did not. MVPA could serve as an alternative or adjunctive approach, particularly in conjunction with depression-focused interventions, to increase resilience, decrease anxiety and depression, and in turn to prevent or attenuate SI in adolescents with ADHD.

**Supplementary Information:**

The online version contains supplementary material available at https://doi.org/10.1007/s10803-025-06809-9.

## Introduction

Attention-deficit/hyperactivity disorder (ADHD) is a common neurodevelopmental disorder characterized by inattention, hyperactivity, and impulsivity (American Psychiatric Association, 2013), which generally develops during childhood and persists into adolescence and adulthood. It is estimated that 5% of children and adolescents have ADHD (Sayal et al., 2018). More importantly, ADHD increases the likelihood of suicidal spectrum behaviors (Septier et al., 2019), with a 4.7-fold higher rate of suicidal behavior than those without ADHD (Fitzgerald et al., 2019). Particularly, 10-19-year-old adolescents with ADHD had a 6.8 times higher rate of suicidal behaviors than their healthy peers (Fitzgerald et al., 2019). Suicide among adolescents with ADHD requires urgent public attention.

Suicide is a serious global public health problem. Each year, more than 700,000 people die from suicide globally, with one person every 40 s (Lovero et al., 2023). It is the third leading cause of death in adolescents, and the fourth leading cause of mortality among 15–29-year-olds worldwide (Lovero et al., 2023). Suicide attempts are the strongest risk factor for suicide (Lovero et al., 2023), with a 12-month risk of suicide of 1.6% and a 5-year risk of suicide of 3.9% after the onset of a suicide attempt (Carroll et al., 2014). Suicidal ideation (SI), which refers to ‘thoughts about taking action to end one’s life’ (Turecki & Brent, 2016), acts as a precipitant for suicide attempts (Bridge et al., 2006). Individuals who reported SI had a high risk of attempting suicide, particularly within the first year of the onset of SI (Nock et al., 2008). Among adolescents, one-third of suicidal ideators develop a plan for a suicidal attempt (Nock et al., 2013). Decreasing SI, therefore, is important for preventing suicide attempts and suicidal behavior.

In adolescents, the rate of SI is 12.1% (Nock et al., 2013), while in those with ADHD, the lifetime prevalence of SI is 33.3% (Mulraney et al., 2021). Physical activity (PA) has shown to be a protective factor against SI in a previous meta-analysis, with people classified as active (i.e., meeting international PA guidelines of 150 min per week of at least moderate or 75 min per week of vigorous-intensity physical activity (MVPA)) reporting less SI (Vancampfort et al., 2018). However, only half of the adolescent studies in this meta-analysis found a significant negative association of PA with SI (Vancampfort et al., 2018). Therefore, it is difficult to determine the extent to which PA is associated with SI in adolescents, not to mention adolescents with ADHD who engage in less PA than their healthy peers (Kim et al., 2011), and do not meet the 60 min of daily MVPA recommended by the World Health Organization (WHO) (Mohammadi et al., 2022). There is a pressing need to examine the association of MVPA with SI in adolescents with ADHD to identify the protective role of MVPA and to detect mediators to facilitate PA interventions for preventing and treating SI in this population.

Mental health may mediate the association between MVPA and SI in adolescents with ADHD. A previous meta-analysis highlighted the positive PA-induced effects on mental health problems in children and adolescents with neurodevelopmental disorders, including ADHD (Liu et al., 2024), which were linked to the onset of suicidal behavior (Fergusson et al., 2000). Notably, approximately 50.3% of children and adolescents with ADHD had at least one comorbid mental disorder (İPÇİ et al., 2020). Especially, 13.2% had depressive disorders (İPÇİ et al., 2020). People with ADHD and clinical depression have a 5-fold higher rate of suicidal behavior than those with ADHD but without any psychiatric diagnosis (Fitzgerald et al., 2019). Depression may contribute the most to SI (Turecki & Brent, 2016). A previous study detected the association between MVPA and depression in children with ADHD (Liang et al., 2023), further suggesting the potential mediating effect of depression between MVPA and SI in adolescents with ADHD.

Apart from depression, other psychiatric comorbidities are also prevalent in adolescents with ADHD, such as elevated levels of anxiety (İPÇİ et al., 2020), and perceived stress (Frick et al., 2023), which are precursors to depression mood in suicide models, acting as important risk factors for SI (Turecki & Brent, 2016). MVPA was significantly associated with anxiety and stress symptoms (Liang et al., 2021; Silverman & Deuster, 2014), further indicating that anxiety and stress may form chain mediation paths with depression between MVPA and SI in adolescents with ADHD, respectively. MVPA may also affect SI through resilience, a protective factor against suicide (Regalla et al., 2019). Resilience is defined as a dynamic developmental process to adapt and overcome negative outcomes (Oddo et al., 2018), and is negatively associated with depression in children with ADHD (Liang et al., 2023). Moreover, adolescents with ADHD reported lower resilience when compared with their healthy peers (Seiffer et al., 2022). This evidence indicates that resilience and depression may mediate the association between MVPA and SI in adolescents with ADHD.

**Fig. 1:**
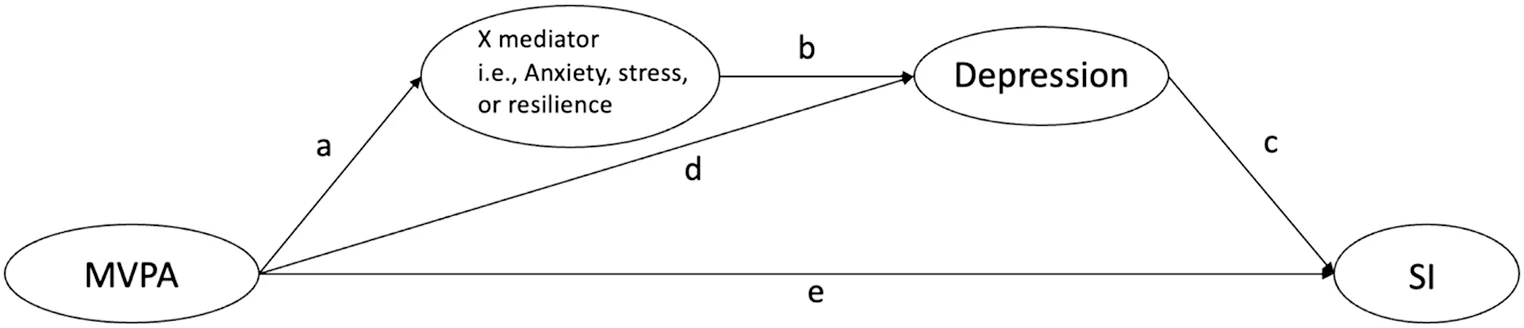
Proposed mediation model with anxiety, stress and resilience mediating the effect of moderate-to-vigorous physical activity (MVPA) on suicidal ideation (SI) with depression, respectively

To date, no studies have examined the association between MVPA and SI in adolescents with ADHD and the mediators of this association. Therefore, this study aimed to examine whether (a) MVPA would be associated with SI in adolescents with ADHD, (b) this association would be mediated by depression (Fig. S1 in Supplement), and (c) anxiety, stress, and resilience would work with depression to constitute chain mediation paths between MVPA and SI (Fig. 1).

## Method

### Participants

One hundred and twenty adolescents aged 12–17 were recruited from eight secondary schools in Hong Kong. The inclusion criteria were: (a) clinically diagnosed with ADHD according to the Diagnostic and Statistical Manual of Mental Disorders, Fifth Edition (DSM-5) by a psychiatrist (American Psychiatric Association, 2013); (b) able to understand and complete the questionnaires independently; (c) receiving education in mainstream secondary schools; (d) absence of other health conditions (e.g., asthma and cardiac disease) that might limit their physical activity participation; and (e) no medication use or medication withdrawal for at least 6 months before the recruitment.

## Measures

### Moderate-to-Vigorous Physical Activity

MVPA data were assessed using accelerometers (wGT3X-BT model; ActiGraph, Pensacola, FL, USA) worn around participants’ waist for seven consecutive days (i.e., five weekdays and two weekend days). Participants wore accelerometers during waking hours, following their daily routines, but except for showering or swimming. A logbook was provided to parents to record their child’s wearing/taking-off time for water activities and the estimated intensity. Consistent with accelerometer wear-time guidelines for good estimates of habitual levels of MVPA in children and adolescents with chronic disease, only data from participants who wore the accelerometers for more than six hours for at least three days (two weekdays and one weekend day) were included (Elmesmari et al., 2017). The time spent at different PA intensities per day was calculated as the mean number of minutes using predefined cut-off points (Evenson et al., 2008).

### Depression, Anxiety, and Stress

The Chinese version of the Depression Anxiety Stress Scale 21 (DASS 21) was used (Wang et al., 2016). It consists of 21 items, divided into three subscales to measure depression, anxiety, and stress over the past week. Each subscale contains seven items, measured on a four-point Likert scale (0 = did not apply to me at all, 1 = some of the time, 2 = a good part of the time, 3 = applied to me very much), with higher scores reflecting higher levels of depression, anxiety, and stress. The Chinese version of the DASS 21 was used to assess depression, anxiety, and stress in children with ADHD in a previous study (Liang et al., 2023). The internal consistency of each subscale was acceptable in this study (depression, α = 0.84; anxiety, α = 0.80; stress, α = 0.83).

### Resilience

The Chinese version of the 25-item Conner-Davidson Resilience Scale (CD-RISC-25) was employed to assess the self-perceived resilience of participants (Yu et al., 2011). Participants were asked to respond to 25 items based on their performance in the previous month. The sample items include “able to adapt to change,” “tend to bounce back after illness or hardship,” and “have a strong sense of purpose.” A five-point Likert scale (0 = not true at all, 4 = true all the time) was used, with higher scores indicating higher levels of resilience. The Chinese version of the CD-RISC-25 has been a reliable and valid measure for assessing resilience in Chinese adolescents (Yu et al., 2011). Its internal consistency was acceptable in the current study (α = 0.98).

### Suicidal Ideation

The Chinese version of the Suicidal Ideation Questionnaire-Junior (SIQ-JR-4) was used to assess the severity of SI among the participants (Zhang et al., 2014). The questionnaire consists of four items: “I thought about killing myself”, “I thought about how I would kill myself”, “I thought about when I would kill myself”, and “I wished I were dead”. Each item is scored on a seven-point likert scale ranging from 0 (I never had this thought) to 6 (almost daily). Higher scores indicate higher SI levels. The SIQ-JR-4 has been demonstrated as a reliable and valid assessment tool for SI in Chinese adolescents (Zhang et al., 2014). The internal consistency of this questionnaire was acceptable (α = 0.97).

### Confounding Structure

We followed previous literature and used a directed acyclic graph (DAG) to identify a minimally sufficient confounder set (Fig. S2 in Supplement). Sex, age, sleep habits (measured with the Children’s Sleep Habits Questionnaire (Li et al., 2007), with higher scores indicating more sleep problems), socioeconomic status (parents’ income interval per month), and dwelling type (i.e., public rental house, home ownership scheme flat, private flat, village house, and others) formed a minimally sufficient confounder set. Descriptive statistics for the minimal confounders are presented in Table 1.

**Table 1:** Demographic and characteristics of the participants (*N* = 60)

Characteristics	mean (SD) / *N*(%)
Sex (males/females)	52/8
Age (years)	14.33 (1.43)
Sleep problems score	47.37 (8.18)
**Socioeconomic status (income per month)**	
HK$10,000–15,000	6 (10.0%)
HK$15,001–20,000	6 (10.0%)
HK$20,001–30,000	12 (20.0%)
HK$30,001–40,000	14 (23.3%)
HK$40,001–50,000	8 (13.3%)
HK$50,001–60,000	3 (5.0%)
HK$60,001–70,000	3 (5.0%)
More than HK$70,000	9 (13.3%)
**Dwelling type**	
Public rental house	28 (46.7%)
Home Ownership Scheme flat	7 (11.7%)
Private flat	15 (25.0%)
village house	6 (10.0%)
Others	4 (6.7%)

### Procedure

The study design complied with the Declaration of Helsinki’s ethical standards and was approved by Joint Chinese University of Hong Kong-New Territories East Cluster Clinical Research Ethics Committee (Reference No.: 2020.451-T & 2021.645). Written informed consent was obtained from the participants and their parents. The participants completed all required questionnaires in the classroom with the assistance of trained research assistants. An accelerometer and a logbook were provided to each participant and his/her parents, and research assistants collected them after seven days.

### Statistical Analysis

The statistical analyses were conducted in R (version 4.3.1, R Foundation for Statistical Computing, Vienna, Austria). Skewness and kurtosis were used to determine whether MVPA, depression, anxiety, stress, resilience, and SI were normally distributed. Bivariate pearson correlations were estimated between the variables. The association between MVPA and SI, and the mediating roles of depression, anxiety, stress, and resilience were examined with the bootstrap method and the adjustment for a minimally sufficient confounder set by using Lavaan package version 0.6–16. Statistical significance was defined as *p* < 0.05.

## Results

### Descriptive Statistics

Sixty participants wore accelerometers for more than six hours for at least three days (two weekdays and one weekend day). Table 1 presents the demographic characteristics of these participants. Descriptive statistics of MVPA, depression, anxiety, stress, resilience, and SI are presented in Table 2. Results indicated mild levels of depression (5.02 ± 3.83) and anxiety (4.77 ± 3.43), and a normal level of stress (6.03 ± 3.55) in participants. Fifteen participants (25%) had SI, with scores ranging from 2 to 24, resulting in a mean score of 2.20 ± 5.28.

**Table 2:** Descriptive statistics and bivariate correlations

Variable	Mean	SD	1	2	3	4	5	6
1. MVPA (mins/day)	21.68	12.85	—					
2. Depression	5.02	3.83	-0.29*	—				
3. Anxiety	4.77	3.43	-0.41**	0.71**	—			
4. Stress	6.03	3.55	-0.39**	0.77**	0.70**	—		
5. Resilience	53.23	19.31	0.35*	-0.47**	-0.19	-0.45**	—	
6. SI	2.20	5.28	-0.26*	0.53**	0.59**	0.52**	-0.19	—

Abbreviation: MVPA, Moderate-to-vigorous physical activity; SI, Suicidal ideation

* *p* < 0.05; ** *p* < 0.01

### Preliminary Analyses

Correlations among MVPA, depression, anxiety, stress, resilience, and SI are presented in Table 2. MVPA was significantly related to depression (*r* = -0.29, *p* = 0.022), anxiety (*r* = -0.41, *p* = 0.001), stress (*r* = -0.39, *p* = 0.002), resilience (*r* = 0.35, *p* = 0.005), and SI (*r* = -0.26, *p* = 0.045). SI was positively correlated with depression (*r* = 0.53, *p* < 0.001), anxiety (*r* = 0.59, *p* < 0.001), and stress (*r* = 0.52, *p* < 0.001), but not with resilience. Significant correlations were also found among depression, anxiety, stress, and resilience, except for the relationship between anxiety and resilience.

### Mediation Analyses

Table 3 shows that MVPA was negatively associated with SI without any covariates (β = -0.260, SE = 0.075, *p* = 0.001). After adjusting for the minimally sufficient confounder set, the association was still significant (adjusted β = -0.265, SE = 0.090, *p* = 0.003). Table 3 also presents the results of the model in Fig. 1, indicating that depression significantly mediated the association between MVPA and SI, with the inclusion of a minimally sufficient confounder set (adjusted indirect effect = -0.160, SE = 0.075, *p* = 0.033). The direct effect of MVPA on SI was not significant, supporting the full mediating effect of depression.

**Table 3:** Bootstrap analysis summary showing the effect of MVPA on SI and the mediation between MVPA and SI by depression in Fig. 1

Path	β [95% CI]	SE	*p*
**Model 1**			
MVPA—SI	-0.260 [-0.408, -0.113]	0.075	0.001
**Model 2†**			
MVPA—SI	-0.265 [-0.442, -0.089]	0.090	0.003
Age—SI	-0.124 [-0.303, 0.055]	0.091	0.174
Sex—SI (ref: Female)			
Male	-0.092 [-0.337, 0.152]	0.125	0.459
Sleep problems—SI	0.023 [-0.285, 0.332]	0.158	0.882
Socioeconomic status—SI	0.058 [-0.351, 0.467]	0.209	0.782
Dwelling type—SI (ref: Public rental house)			
Home Ownership Scheme flat	-0.205 [-0.396, -0.013]	0.098	0.036
Private flat	-0.288 [-0.543, -0.033]	0.130	0.027
Village house	-0.075 [-0.326, 0.176]	0.128	0.558
Others	-0.226 [-0.355, -0.097]	0.066	0.001
**Model 3† (**Fig. 1**)**			
MVPA—Depression (a path)	-0.346 [-0.569, -0.122]	0.114	0.002
Depression—SI (b path)	0.464 [0.204, 0.724]	0.133	0.001
Indirect effect (a × b)	-0.160 [-0.308, -0.013]	0.075	0.033
Direct effect (c path)	-0.105 [-0.315, 0.105]	0.107	0.329

Abbreviation: MVPA, Moderate-to-vigorous physical activity; ref, reference group; SI, Suicidal ideation

Note. † Adjust for the minimally sufficient confounder set

With reference to Fig. 2; Table 4 summarizes the results of the mediation analyses between MVPA and SI by anxiety, stress, resilience, and depression while adjusting for the minimally sufficient confounder set. Anxiety (adjusted indirect effect = -0.159, SE = 0.065, *p* = 0.014), stress (adjusted indirect effect = -0.163, SE = 0.070, *p* = 0.019), and resilience (adjusted indirect effect = -0.088, SE = 0.042, *p* = 0.035) worked with depression to form chain mediation paths between MVPA and SI, respectively.

**Table 4:** Bootstrap analysis summary showing the mediation between MVPA and SI by anxiety, stress, resilience, and depression in fig. 2

Path	Model (X mediator in Fig. 2)												
	Model 4 (Anxiety)†				Model 5 (Stress)†				Model 6 (Resilience)†				
	β [95% CI]	SE	*p*		β [95% CI]	SE	*p*		β [95% CI]	SE	*p*		
MVPA—X mediator (a path)	-0.457 [-0.636, -0.279]	0.091	0.000		-0.454 [-0.649, -0.259]	0.100	0.000		0.443 [0.227, 0.658]	0.110	0.000		
X mediator—Depression (b path)	0.750 [0.559, 0.940]	0.097	0.000		0.771 [0.623, 0.920]	0.076	0.000		-0.427 [-0.639, -0.215]	0.108	0.000		
Depression—SI (c path)	0.464 [0.198, 0.729]	0.135	0.001		0.464 [0.201, 0.727]	0.134	0.001		0.464[0.203, 0.725]	0.133	0.001		
MVPA—Depression (d path)	-0.003 [-0.192, 0.186]	0.096	0.975		0.005 [-0.197, 0.206]	0.103	0.808		-0.157 [-0.382, 0.069]	0.115	0.174		
Indirect effect 1 (a × b × c)	-0.159 [-0.286, -0.032]	0.065	0.014		-0.163 [-0.299, -0.026]	0.070	0.019		-0.088 [-0.169, -0.006]	0.042	0.035		
Indirect effect 2 (c × d)	-0.001 [-0.089, 0.086]	0.045	0.975		0.002[-0.092, 0.096]	0.048	0.965		-0.073[-0.187, 0.042]	0.058	0.213		
Direct effect (e path)	-0.105 [-0.315, 0.106]	0.107	0.329		-0.105 [-0.315, 0.105]	0.107	0.329		-0.105 [-0.315, 0.105]	0.107	0.329		

Abbreviation: MVPA, Moderate-to-vigorous physical activity; SI, Suicidal ideation

Note. X mediator represents anxiety, stress, or resilience, shown in Fig. 2

† Adjust for the minimally sufficient confounder set

After adding significant mediators (i.e., anxiety, stress, resilience, and depression) to the model shown in Fig. S3 in Supplement, the results presented in Table 5 indicate that the chain mediation paths through anxiety and depression (adjusted indirect effect = -0.104, SE = 0.049, *p* = 0.032), and resilience and depression (adjusted indirect effect = -0.060, SE = 0.030, *p* = 0.043) were significant. Stress did not act as a mediator in this context. Anxiety and depression explained 38.2% of the variance (calculated as β for adjusted indirect effect / β for adjusted total effect) in the association between MVPA and SI, while resilience and depression explained 22.1%.

**Table 5:** Bootstrap analysis summary showing the mediation between MVPA and SI by anxiety, stress, resilience, and depression in fig. 3

Path	Model 7†			
	β [95% CI]	SE	*p*	Proportions mediated
MVPA—Anxiety (a path)	-0.457 [-0.636, -0.279]	0.091	0.000	
Anxiety—Depression (b path)	0.547 [0.211, 0.884]	0.172	0.001	
MVPA—Stress (c path)	-0.454 [-0.649, -0.259]	0.100	0.000	
Stress—Depression (d path)	0.443 [0.052, 0.834]	0.199	0.026	
MVPA—Resilience (e path)	0.443 [0.227, 0.658]	0.110	0.000	
Resilience—Depression (f path)	-0.328[-0.556, -0.100]	0.116	0.005	
MVPA—Depression (g path)	0.201 [0.004, 0.398]	0.101	0.046	
Depression—SI (h path)	0.416 [0.173, 0.660]	0.124	0.001	
Indirect effect 1 (a × b × h)	-0.104 [-0.200, -0.009]	0.049	0.032	38.2%
Indirect effect 2 (c × d × h)	-0.084 [-0.188, 0.003]	0.053	0.115	
Indirect effect 3 (e × f × h)	-0.060 [-0.119, -0.002]	0.030	0.043	22.1%
Indirect effect 3 (g × h)	0.084 [-0.011, 0.178]	0.048	0.083	
Direct effect (i path)	-0.108 [-0.322, 0.107]	0.109	0.325	

Abbreviation: MVPA, Moderate-to-vigorous physical activity; SI, Suicidal ideation

† Adjust for the minimally sufficient confounder set

## Discussion

The current study investigated the association of MVPA with SI in adolescents with ADHD and examined the mediating effects of depression, anxiety, stress, and resilience in this association. Our findings indicated that MVPA was negatively associated with SI, regardless of whether a minimally sufficient confounder set was included. After adjusting for the minimally sufficient confounder set, anxiety, stress, and resilience, combined with depression, formed three chain mediation paths between MVPA and SI, respectively. In the integrative model that included all significant mediators and was adjusted for the confounder set, two significant chain mediation paths formed by anxiety, resilience, and depression were found, whereas the indirect effect across stress and depression was not significant.

Consistent with a previous study (Mohammadi et al., 2022), participants in the current study engaged in daily MVPA for an average of 21.68 min, which fell short of WHO’s PA recommendation. More importantly, we confirmed a significant association between MVPA and SI in adolescents with ADHD. Especially, depression played as a main mediator between MVPA and SI, working with stress, anxiety, and resilience. A strong relationship between psychopathology, particularly major depressive disorder, and suicide risk has been reported (Bridge et al., 2006). In addition to the clinical diagnosis, depressed mood is also frequently accompanied by SI (Bridge et al., 2006), consistent with our results. The high overlap of biological mechanisms of depression and suicidality may give explanation (Brundin et al., 2017). Robust evidence has shown the PA-induced benefits for alleviating depression in adolescents (Recchia et al., 2023), as well as in those with ADHD (Zang, 2019). However, when using PA interventions to treat depression in adolescents, the intensity should be carefully managed and maintained at least of a moderate level (Gu, 2022). The negative association of MVPA with depression in adolescents with ADHD in the current study not only supported this view but was also identified in children with ADHD (Liang et al., 2023). We further found the mediating effect of depression between MVPA and SI. A similar role of depression was also identified between PA and SI in healthy adolescents (Taliaferro et al., 2010). Particularly, the full mediation effect of depression observed in the current study partially supported the view that depression accounted for most of SI (Turecki & Brent, 2016).

ADHD was considered associated with dysregulation of the hypothalamus-pituitary-adrenal (HPA) axis (Ma et al., 2011). It has been examined that individuals who manifest suicidal behavior have an inappropriate hyperactivity of HPA axis (Turecki et al., 2012). HPA axis is the primary neuroendocrine response facilitating adaptation to stress (Aguilera, 2011). The dysregulation of HPA axis, such as a higher cortisol awakening response, is further associated with pathophysiology of anxiety and depression (Wardenaar et al., 2011). Exercise is a promising approach to buffer overall cortisol secretion by shortening the cortisol response (Chen et al., 2017). However, the intensity should be well-organized. A previous study found that more vigorous exercise could reduce the cortisol response and accelerate recovery from a subsequent psychosocial stressor (Caplin et al., 2021). Therefore, it is plausible that MVPA may affect SI through the regulation of the HPA axis, mediated by stress and depression in sequence.

Anxiety and depression are often comorbid, and it has been found that individuals characterized by high levels of anxiety are likely to experience SI (Bridge et al., 2006). Evidence showed that anxiety, depression, and suicidal attempts were all associated with decreased brain-derived neurotrophic factor (BDNF) (Turecki et al., 2012). Also, BDNF has been implicated with the pathophysiology of ADHD (de Lucca et al., 2023). Exercise is confirmed an approach to increasing the level of BDNF (Gubert & Hannan, 2021). However, it has been reported that only moderate- and high-intensity PA could increase BDNF in youth, suggesting that at least 50% VO_2MAX_ is required to induce benefits for BDNF (Heinze et al., 2021). Previous study found that children and adolescents with ADHD also displayed an elevated sympathetic reactivity (Morris et al., 2020). The increased sympathetic activity was, in turn, linked with depressive symptoms and SI (Alacreu-Crespo et al., 2024; Määttänen et al., 2019). The children’s and adolescents’ participation in MVPA would decrease the sympathetic nerve activity (Chen et al., 2022), and lead to the repeated exposure to the anxiety-related interoceptive stimuli, such as similar bodily sensation, resulting in an adaptation in sympathetic activity which, and therefore alleviate the anxiety, especially physical symptoms of anxiety (de Coverley Veale, 1987). The evidence supports the mediating effects of anxiety and depression on the relationship between MVPA and SI in adolescents with ADHD.

Resilience is correlated to psychological ill-being and SI in adolescents (Hu et al., 2015; Park et al., 2010). Meta-analytical findings showed that PA interventions were beneficial to resilience in adolescents (Andermo et al., 2020). A previous study detected the mediating effect of resilience between MVPA and depression in children with ADHD (Liang et al., 2023), in line with our findings. By increasing PA volume, adolescents would gain more social support and learn coping skills (Lubans et al., 2016), which contribute to the development of resilience against depression and SI (Wang et al., 2022). PA also benefits hormonal stress responsive systems, minimize excessive inflammation, and enhance growth factor expression and neural plasticity, which are recognized as biological mechanisms between PA and resilience (Oddo et al., 2018). Moreover, children and adolescents who receive more years of ADHD treatment are more likely to be resilient to depression (Oddo et al., 2018), while MVPA has ready been treated as an alternative treatment for ADHD in children and adolescents (Seiffer et al., 2022).

Interestingly, after adding all the variables to the model, anxiety, resilience, and depression still significantly mediated the association of MVPA with SI, while stress did not. Stress might be the precursor to anxiety and resilience, as both two factors were related to the stress responsive systems, as mentioned above.

To the best of our knowledge, this was the first study to examine the association between MVPA and SI in adolescents with ADHD and the potential mediators. Our study had several limitations. First, the small sample size may lead to underpowered results. Second, sex may have an impact on SI. However, an unbalanced sex distribution was observed in this study, making it challenging to examine the moderating effects of sex. Third, the Diagnostic and Statistical Manual of Mental Disorders, Fifth Edition (DSM-5) (American Psychiatric Association, 2013), defines three subtypes of ADHD clinical presentations based on symptom count: predominantly hyperactive–impulsive subtype (ADHD-HI), predominantly inattentive subtype (ADHD-I), and a combined subtype (ADHD-C), which may affect the results. Moreover, the severity of ADHD symptoms may also influence the outcomes. Given the limited sample size of 60 participants, it is difficult to explore the moderating effects of both ADHD subtypes and severity. Future studies are encouraged to do so, as they would have significant clinical implications. Overall, the present study suggested that MVPA could serve as an alternative or adjunctive approach, particularly in conjunction with depression-focused interventions, to increase resilience, decrease anxiety and depression, and in turn to prevent or attenuate SI in adolescents with ADHD.

## Electronic supplementary material

Below is the link to the electronic supplementary material.
