# Supplementary material for: Moderate-to-Vigorous Physical Activity and Suicidal Ideation in Adolescents with Attention Deficit/Hyperactivity Disorder: the Mediating Effects of Mental Health
Source: J Autism Dev Disord. 2025 Apr 20;56(10):3765–73. doi: 10.1007/s10803-025-06809-9 (PMC13538217; doi:10.1007/s10803-025-06809-9)
Supplement: Supplementary file 1 [file 10803_2025_6809_MOESM1_ESM.docx]

**Supplemental Content**

**Fig S1.** Proposed mediation model with depression mediating the effect of moderate-to-vigorous physical activity (MVPA) on suicidal ideation (SI).

**Fig S2.** Final directed acyclic graph (DAG) for the association between MVPA and SI with proposed mediators.

**Fig S3.** Proposed mediation model with depression, anxiety, stress, and resilience mediating the effect of moderate-to-vigorous physical activity (MVPA) on suicidal ideation (SI).

**Fig S1.** Proposed mediation model with depression mediating the effect of moderate-to-vigorous physical activity (MVPA) on suicidal ideation (SI).

**Fig S2.** Final directed acyclic graph (DAG) for the association between MVPA and SI with proposed mediators.

The Figure shows that sex, age, sleep habits, socioeconomic status (SES), and dwelling type formed a minimally sufficient confounder set (colored pink) to estimate the effect of moderate-to-vigorous physical activity (MVPA) on suicidal ideation (SI) in adolescents with ADHD. Potential causal paths are shown in green and non-causal paths are shown in pink.

**Fig S3.** Proposed mediation model with depression, anxiety, stress, and resilience mediating the effect of moderate-to-vigorous physical activity (MVPA) on suicidal ideation (SI).
